# Peutz–Jeghers Syndrome and the Role of Imaging: Pathophysiology, Diagnosis, and Associated Cancers

**DOI:** 10.3390/cancers13205121

**Published:** 2021-10-13

**Authors:** Sergio Klimkowski, Mohamed Ibrahim, Juan J. Ibarra Rovira, Mohamed Elshikh, Sanaz Javadi, Albert R. Klekers, Abdelraham A. Abusaif, Ahmed W. Moawad, Kamran Ali, Khaled M. Elsayes

**Affiliations:** 1 Department of Abdominal Imaging, The University of Texas MD Anderson Cancer Center, Houston, TX 77555, USA; jjibarra@mdanderson.org (J.J.I.R.); Sanaz.Javadi@mdanderson.org (S.J.); ARKlekers@mdanderson.org (A.R.K.); Abdelrahman.abusaif@mercyhealth.org (A.A.A.); 2 Department of Diagnostic and Interventional Radiology, University of Kansas-Wichita, Wichita, KS 67214, USA; mibrahim2@kumc.edu (M.I.); kali@kumc.edu (K.A.); 3 Department of Diagnostic and Interventional Radiology, The University of Texas Medica Branch, Galveston, TX 77555, USA; moelshik@utmb.edu; 4 Department of Diagnostic and Interventional Radiology, Mercy Catholic Health System, Darby, PA 19023, USA; ahmedw.moawad@mercyhealth.org

**Keywords:** Peutz-Jeghers Syndrome, PJS, Peutz-Jeghers Syndrome imaging

## Abstract

**Simple Summary:**

The Peutz-Jeghers Syndrome is a rare autosomal dominant syndrome characterized by mucocutaneous pigmentations, multiple gastrointestinal hamartomatous polyps, and an elevated risk of malignancy. Awareness of various Peutz-Jeghers Syndrome imaging patterns, associated malignancies, and their complications is crucial for accurate imaging interpretation and patient management. In this manuscript, we provide an overview of this condition, associated malignancies, and imaging surveillance protocols.

**Abstract:**

The Peutz-Jeghers Syndrome (PJS) is an autosomal dominant neoplastic syndrome defined by hamartomatous polyps through the gastrointestinal tract, development of characteristic mucocutaneous pigmentations, and an elevated lifetime cancer risk. The majority of cases are due to a mutation in the STK11 gene located at 19p13.3. The estimated incidence of PJS ranges from 1:50,000 to 1:200,000. PJS carries an elevated risk of malignancies including gastrointestinal, breast, lung, and genitourinary (GU) neoplasms. Patients with PJS are at a 15- to 18-fold increased malignancy risk relative to the general population. Radiologists have an integral role in the diagnosis of these patients. Various imaging modalities are used to screen for malignancies and complications associated with PJS. Awareness of various PJS imaging patterns, associated malignancies, and their complications is crucial for accurate imaging interpretation and patient management. In this manuscript, we provide a comprehensive overview of PJS, associated malignancies, and surveillance protocols.

## 1. Introduction

**Figure 1:**
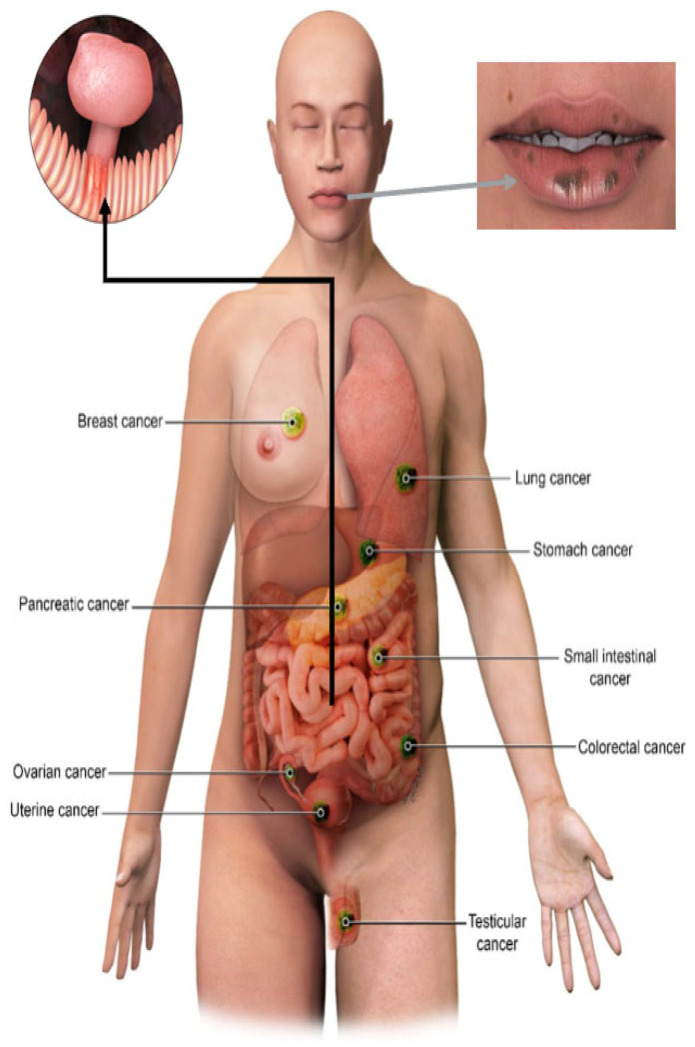
Manifestations of Peutz-Jeghers Syndrome include characteristic mucocutaneous pigmentations, hamartomatous gastrointestinal polyps, and an elevated risk of malignancy.

**Figure 2:**
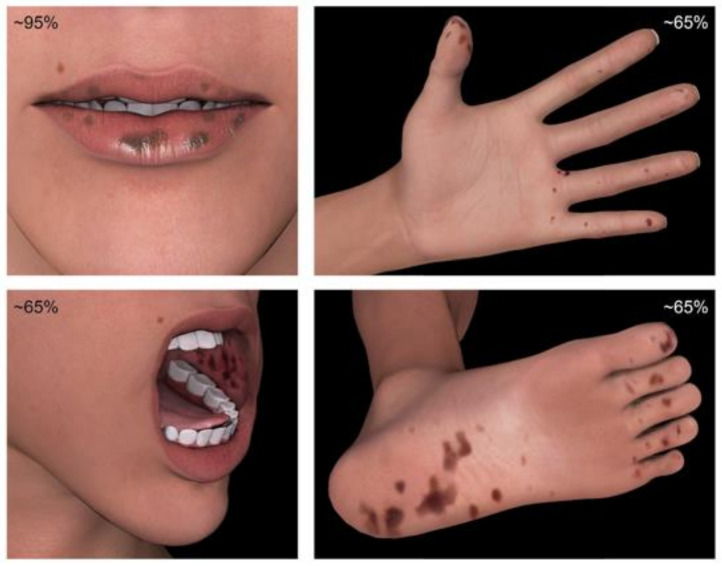
The mucocutaneous pigmentations of PJS.

The Peutz-Jeghers Syndrome (PJS) is a rare inherited autosomal dominant syndrome characterized by mucocutaneous pigmentations, multiple gastrointestinal hamartomatous polyps, and an increased risk of malignancies (Figure 1 and Figure 2) [1]. The first descriptions of this disorder date back to the late 1800s by Dr. Conner and later in the 1920s by Dr Peutz. However, a combination of intestinal polyposis and mucocutaneous pigmentations was first described as a distinct entity in 1949 by Dr. Jeghers [2]. Brewer et al. coined the eponym “Peutz-Jeghers Syndrome” in 1954. The estimated incidence of PJS ranges from 1:50,000 to 1:200,000 births with no gender or racial predilection [3,4]. Most of the cases result from a mutation in the STK11 tumor suppressor gene. As a result, PJS is associated with an increased risk of malignancies resulting in significant morbidity and mortality. The most common cancers in this patient population include gastrointestinal, genitourinary, breast, and lung malignancies (Figure 1). This review article will discuss the common clinical manifestations of Peutz-Jeghers syndrome, diagnostic criteria, genetics, associated malignancies, and requisite imaging screening protocols, including the National Comprehensive Cancer Network (NCCN) recommendations.

## 2. Diagnostic Criteria

A clinical diagnosis of Peutz-Jeghers syndrome is made when one of the following criteria are met [1,5]:

1. Two or more pathologically confirmed Peutz-Jeghers polyps;
2. Any Peutz-Jeghers polyps and family history of Peutz-Jeghers;
3. Characteristic mucocutaneous pigmentations involving the mouth, lips, nose, eyes, genitalia or fingers with a family history of PJS;
4. Any Peutz-Jeghers polyps in patients with characteristic mucocutaneous pigmentations.

Moreover, genetic testing, detailed clinical history (including detailed information from childhood), and familial history play a key role in the diagnosis. Relatives of the confirmed PJS cases should be genetically tested.

## 3. Genomics and Pathophysiology

The majority of cases are due to a mutation in the STK11 gene (also known as the LKB1 gene) located at 19p13.3. This gene encodes the tumor suppressor serine/threonine kinase and 50–90% of PJP cases are due to mutations in this enzyme resulting in the loss of the kinase function [5,6,7]. STK11/LKB1 is involved in multiple signaling pathways that control cell-cycle and cell proliferation, including processes such as apoptosis and RAS induced cell transformation. Most of the detected mutations are either truncating or missense mutations. Although, up to 30% of mutations may be large deletions which could be missed by sequencing alone. Therefore, the genetic analysis of these patients should include both gene sequencing and assessment for large deletions. A small percentage of patients that meet the diagnostic criteria for PJS show no identifiable mutations in STK11. This suggests that another, yet unidentified, gene focus maybe involved [8]. Mutations in SPK11 are inherited in the autosomal dominant fashion with variable penetrance, resulting in varied phenotypes. Although, 25% of cases occur de novo without a known prior family history of this disorder. Therefore, it is essential to carefully evaluate the patient’s family before making a presumption of de novo mutation.

Missense mutations tend to have a later age of presentation (onset of polyps or other symptoms) than truncating mutations or cases where no mutations are identified. Amos et al. found the onset of symptoms to be 23, 13, and 15 years of age for missense mutations, truncation mutations or unknown mutations, respectively [5]. However, no statistically significant difference was found when evaluating the risk of intussusception (major factor in morbidity and mortality of this disorder) and mutation type [9].

Predictably, mutations in a tumor suppressor gene result in an increased lifetime cancer risk. Utsunomiya et al. studied the natural history of this disorder in Japan and found increased risk of cancer mortality. However, this elevated risk comes with better survival than patients with familial adenomatous polyposis [3]. Patients with PJS have 81–93% cumulative lifetime risk of developing any malignancy [10,11]. Cancer-related mortality accounts for up to 66% of the overall mortality in PJS with an average age of cancer diagnosis of 42 years compared to 66 years of age in the general population [12].

In the past, the histopathological assessment of polyps led to the unclear assessment of cancer risk, with some authors demonstrating a lack of correlation between the location of primary GI malignancy and polyps. Numerous papers demonstrate a well-established increased risk of gastrointestinal malignancy in PJS. Although, a specific origin of cancer within the GI tract remains less clear. Large polypoid hamartomas can have foci of adenomatous change and some studies have demonstrated evidence of hamartomatous-adenomatous-carcinomatous histologic progression in gastric, small bowel, and colonic polyps in patients with PJS [3,13,14,15,16,17]. Additionally, some authors found possible evidence of direct hamartoma/carcinoma sequence [18], while others have proposed that polyps found in PJS have no malignant potential [19]. This hypothesis suggests that the malignant transformation within a polyp is a rare event and that PJS polyps actually represent abnormal prolapsed mucosal surfaces due to changes in cellular polarity, related to the STK11 gene mutation (rather than a true hamartoma). The increased cancer risk could be related to a background of cellular instability, presumably related to the accelerated pathway of conventional neoplastic mechanisms [19].

## 4. Clinical Manifestations

The mucocutaneus pigmentations seen in PJS are due to characteristic macules, typically seen on the mouth, buccal mucosa, and genital and perianal mucosal surfaces. Distinctive features of PJS include perioral pigmentations from perioral freckles with darker color, dense claustration, and crossing of the vermillion border (Figure 2). Moreover, other regions such as fingers, soles, palms, and periorbital regions are commonly affected [20,21]. This abnormal pigmentation manifests during infancy and tends to fade during adolescence. Although, pigmented lesions in the mouth may persist into adulthood [20].

**Figure 3:**
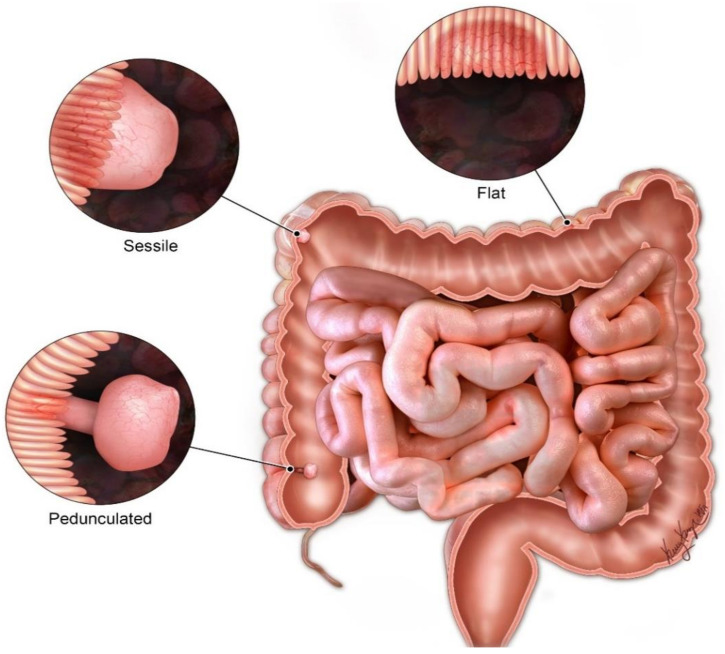
Different types of GIT polyps in PJS.

**Figure 4:**
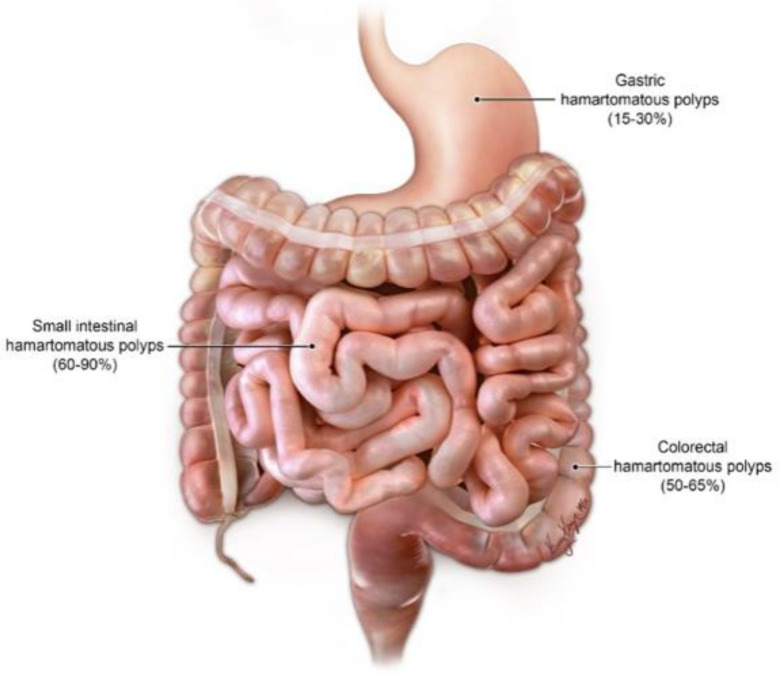
Distribution of GIT polyps in PJS.

The GI symptoms tend to present early with the median age of symptom onset being 13 years old. Roughly, 50% of patients will be symptomatic by 20 years of age [22]. Although, a small percentage of individuals with PJS may develop symptoms later in life or have vague nonspecific symptoms, such as abdominal pain. The common presenting symptoms are usually related to gastrointestinal problems, such as intussusception or obstruction. Additionally, rectal bleeding and anemia can be seen [20]. The GI symptoms are related to the presence of hamartomatous polyps predominately found in the small bowel rather than the colon (in distinction to other hamartomatous polyposis syndromes). Polyps can be flat, sessile or pedunculated (Figure 3). Hamartomatous histology refers to fork-like extensions of smooth muscle into the lamina propria. The highest numbers of polyps tend to occur in the jejunum followed by ileum and duodenum (Figure 4). However, 25–30% of patients with PJS will have polyps in the colon and the stomach. The rectum is the least affected part of the GI tract with PJS hamartomatous polyps. These polyps usually vary in size from 1 mm to 3 cm and are usually present in the second or third decade of life. Patients with PJS may also develop hamartomatous polyps in locations other than the GI tract, e.g., respiratory and genitourinary tracts.

**Figure 5:**
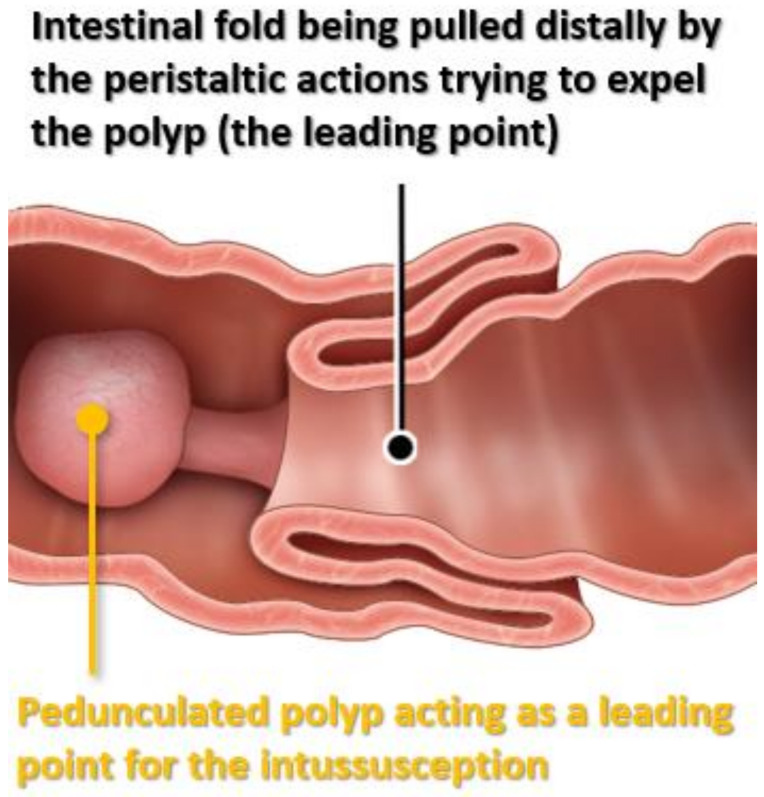
Diagrammatic illustration of PJS GIT polyp as the leading point of intussusception.

**Figure 6:**
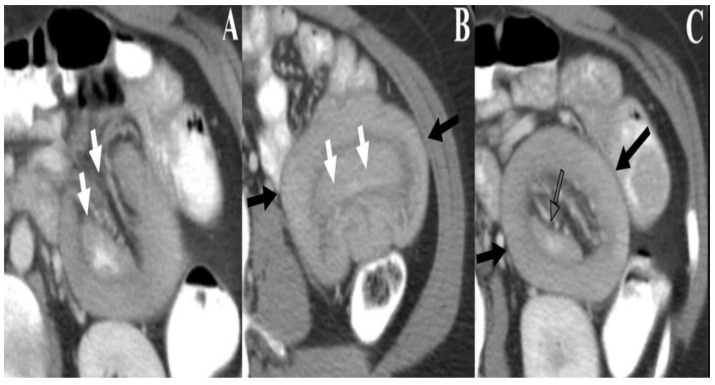
A 43-year-old patient with PJS and intestinal polyposis presented to the ER with abdominal pain. Axial contrast enhanced CT images of the abdomen (**Panels A**–**C**) demonstrate jejuno-jejunal intussusception, with telescoping of the mesenteric fat and loops of proximal jejunum (intussusceptum, white arrows) into the more distal jejunum (intussuscipiens, black arrow). An intestinal polyp was found to be the lead point and cause of intussusception (clear arrow).

Intussusception occurs in around 70% of PJS patients with the intestinal polyps acting as lead points (Figure 5). Unlike most pediatric intussusceptions which occur frequently in the ileocecal area, the PJS-related intussusceptions are usually ileo-ileal or jejuno-jejunal (Table 1). Thirty percent of all the PJS mortalities are related to intussusceptions (Figure 5 and Figure 6). Both small and large bowel polyps tend to be pedunculated and stomach polyps tend to be sessile. The large polyp size and pedunculated morphology contribute to the recurrent intussusception and obstructive symptoms, frequently requiring operative management [20]. Bartholomew et al. described the nonneoplastic hamartomatous histology of these polyps in 1957 [23]. Intestinal polyps can also contain mucin cysts, which can enlarge and result in enteritis cystica profunda with an obstruction necessitating a surgical intervention [24].

Aside from the morbidity and mortality related to the GI tract obstruction, GI and non-GI malignancies are the major clinical concerns when managing this patient population. The most common cancers in this patient are GI, GU, breast, and lung malignancies.

**Table 1:** Difference between usual and PJS-associated intussusception.

	Usual Intussusception	PJS-Associated Intussusception
Cause	ldiopathic, viralinfection, Meckel’s diverticulum	Hamartomatous Polyp (leading point)
Age	First 2 years of life	Usually > 10 years old
Site	Usually ileocecal	Usually jejuno-jejunal or ileo-ileal
Treatment	Usually air or saline enema is enough	Usually surgery or enteroscopy
Prognosis	Excellent	Responsible for up to 30% of PJS-associated mortality

## 5. Diagnostic Challenges and Differential Considerations

PJS shares some of its hallmark features with other polyposis disorders, such as the Bannayan-Riley Ruvalcaba syndrome (BRRS), Cowden syndrome (CS), Laugier-Hunziker syndrome (LHS), and Juvenile Polyposis syndrome (JPS).

The Bannayan-Riley Ruvalcab and Peutz-Jeghers syndromes can be difficult to distinguish, since they share common clinical manifestations. Both are characterized by hamartomas in the intestines and pigmented spots in genital regions. However, BRRS can be distinguished from PJS by the presence of macrocephaly and developmental delays. Genetically, a germline mutation of the phosphatase and tensin homolog in the chromosome 10 tumor suppressor gene (PTEN) is present in patients with BRRS and CS [22,25,26,27].

The Laugier-Hunziker syndrome is also characterized by perioral macular pigmentations and polyps. However, unlike PJS, the intestinal polyps have a later onset and usually appear in adulthood [28]. This syndrome is benign and typically the diagnosis is based on exclusion [29]. Therefore, it is crucial to differentiate this disorder from other mucocutaneous polyposis syndromes that may require surveillance and/or additional medical management.

The Peutz-Jeghers syndrome tends to present with a small bowel obstruction. On the other hand, Juvenile Polyposis typically presents with rectal bleeding. JPS is due to mutations in SMAD4 and BMPR1A genes (not STK11 as is the case with PJS).

## 6. Common Malignancies in PJS and Imaging Screening Protocols

Malignancies in the Peutz-Jegher syndrome are broadly subdivided into gastrointestinal and non-gastrointestinal cancers. Gastrointestinal cancers are the most common malignancies in PJS patients, accounting for up to two thirds of malignancies in this population. These malignancies predominately include colorectal, small bowel, esophageal, and gastric cancers [10]. Malignancy specific imaging screening protocols based on the NCCN recommendations are summarized below (Table 2 and Table 3).

### 6.1. Gastrointestinal Malignancies

**Figure 7:**
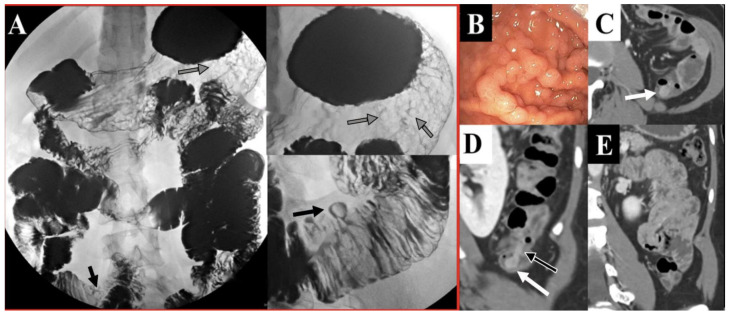
A 23-year-old female with known PJS. (**Panel A**) Upper GI series with barium contrast. Multiple filling defects throughout the stomach (gray arrows, upper figure) and jejunum (black arrows, lower figure) are characteristic of PJS. Presence of these polyps was confirmed by upper endoscopy (**Panel B**). (**Panels C** and **D**) Contrast-enhanced CT scan with enhancing polypoid lesions (white arrows) in the descending colon (axial section in **panel C**, coronal images in **panels D** and **E**). One of the polyps appears to have a thin stalk (black arrow with white border).

Colorectal cancer is the most common gastrointestinal malignancy in PJS patients. The risk of developing colorectal cancer is reported to be as high as 39% and increases with age [10]. Screening protocols for GI cancers include traditional endoscopy and/or video capsule endoscopy for a proper visualization of the small intestine. Moreover, CT or MR enterography can be used as alternative screening modalities [30,31]. Furthermore, the fluoroscopic GI series can be performed in patients who cannot or do not desire endoscopy (Figure 7). Capsule endoscopy is more sensitive in the detection of polyps than small bowel fluoroscopic studies [32]. However, this approach underestimates the number of polyps. At least 20% or more additional polyps have been identified on the enteroscopy when compared to the capsule endoscopy alone [33]. A baseline screening with upper endoscopy and colonoscopy should be performed at 8 years of age. If polyps are present, then patients should be routinely screened with repeat endoscopy every 2 to 3 years. If the initial screening reveals no polyps, then endoscopic screening can resume at the age of 18 and repeat every 2 to 3 years (Table 1 and Table 2) [12,34].

### 6.2. Pancreatic Cancer

**Figure 8:**
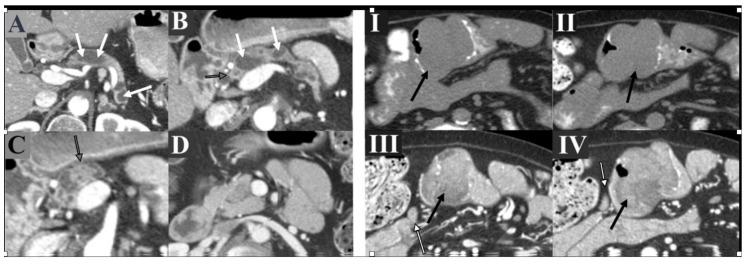
A 35-year-old female patient with known PJS. (**Left Panel**) Annual screening of CT abdomen (Subpanels **A**–**D**) showed diffuse dilatation of the main pancreatic duct (white arrows) with hypoattenuating polypoid lesions in the pancreatic region (black-bordered gray arrows). Biopsy with histopathological examination showed intra-ductal mucinous neoplasm (benign tumor). (**Right Panel**) Bulky soft tissue mass (black arrows) was incidentally identified along the proximal small bowel (Subpanels **I**, **II**) with heterogeneous enhancement (Subpanels **III**, **IV**) and regional lymphadenopathy (white arrows). Surgical excision of this mass revealed mucinous adenocarcinoma of the small bowel with lymph node involvement.

Pancreatic cancer is also a commonly associated malignancy with PJS. The risk of developing pancreatic cancer is reported to be as high as 36–40% with a mean presentation age of 59 years old [35]. Screening should ideally start around the age of 25–35. Magnetic resonance cholangiopancreatography (MRCP) or endoscopic ultrasonography (EUS) should be used for the initial screening and repeated every 2–3 years (Figure 8) [11,12,36]. EUS is more invasive and may be more sensitive in experienced hands (although very operator dependent) [37].

### 6.3. Gynecologic Cancers

Gynecologic malignancies are common with the Peutz-Jegher syndrome. A lifetime risk of developing ovarian cancer in this patient population is about 21% [11]. The average age for developing ovarian cancer in PJS patients is 28 years of age [11]. These elevated risks are comparable to cancer risks in other hereditary conditions, such as patients with BRCA 1 and 2 mutations. Therefore, the PJS patients should follow the screening guidelines already established for those high-risk patients. The experts recommend an annual screening transvaginal ultrasound and serum CA-125 beginning at the age of 25. However, currently, there is no established evidence to support any imaging screening modality for gynecologic cancer in PJS patients. These patients are prone to developing sex cord tumors with annular tubules (SCAT), a characteristic feature of PJS. Additionally, over one third of women diagnosed with SCAT have the Peutz-Jegher syndrome. Screening for cervical cancer should be the same as for the general population. The patients with PJS tend to develop adenoma malignum (also known as minimal deviation adenocarcinoma or MDA), a rare variant of cervical adenocarcinoma. Sonographically, this tumor appears as a multilocular grape-like cystic clusters within the cervix and may contain heterogenous solid components. If not careful, this appearance can be confused with large complex Nabothian cysts [38].

### 6.4. Breast Cancer

Breast cancer is the second most common malignancy associated with PJS, affecting 32–54% of these patients. The mean age of breast cancer diagnosis is 37 years of age (ranges 19 to 48 years of age) [10,11]. These risks are on par with other high-risk syndromes, such as BRCA1/BRCA2 mutations (40 to 85% of lifetime risk) [39,40,41]. Screening guidelines based on expert opinion and developed by the Cancer Genetics Studies Consortium (organized by the National Human Genome Research Institute) were recently adapted by the National Comprehensive Cancer Network. However, true efficacy of these recommendations in this patient population remains unknown. The high-risk screening comprises monthly self-examinations starting at 18 years of age and a semi-annual breast clinic evaluation. The annual mammography should be started at 25 years of age but is often based on the family history of the earliest age of onset. The MRI maybe performed in lieu of mammography in cases of technical limitations or to decrease the lifetime radiation dose. Prophylactic mastectomies and counseling can be discussed. However, no guidelines exist to guide the management in this patient population.

### 6.5. Testicular Cancer

Testicular sonography is only recommended in cases of testicular abnormalities, which are found on physical exam or in cases of precocious puberty. The majority of the testicular cancers in PJS are Sertoli cell tumors with 9 years old being the average age at diagnosis [11].

### 6.6. Lung Cancer

**Figure 9:**
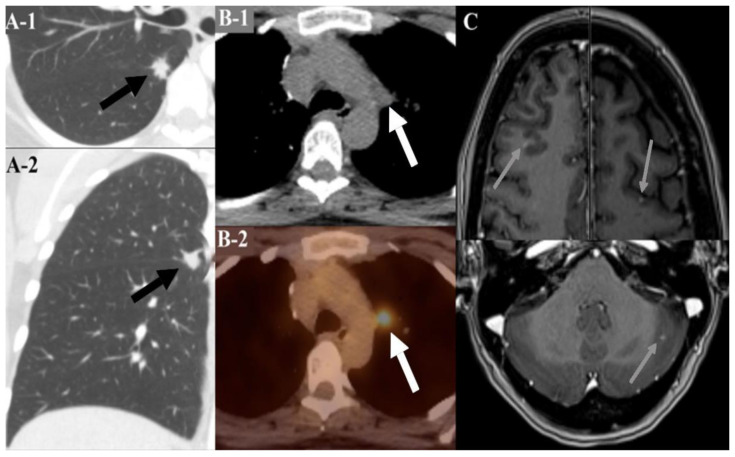
(**Panel A**) Routine CT chest showed a pulmonary mass with speculated irregular borders (black arrows) at the major fissure (axial in **A-1**, and coronal in **A-2**). Histopathological examination showed lung adenocarcinoma. (**Panel B**) another CT chest (**B-1**) and PET/CT image (**B-2**) with a moderately FDG-avid aorto-pulmonary window lymph node (white arrows) with SUV = 6.3. A further work-up demonstrated the metastatic lesions seen on the brain MRI (gray arrows in **panel C**).

Lim et al. found a 7% risk of lung cancer by the age of 60 (out of 240 patients with STPK11 mutations). The corresponding risk for the general population at age 60 is approximately 1%. Therefore, this represents a 7-fold increase in risk [42]. However, no expert consensus or published guidelines exist on screening these patients with the STK11 mutation for lung cancer (aside from smoking-related guidelines). Pulmonary lesions in this patient population are usually discovered incidentally or when performed during a metastatic work-up (Figure 9).

## 7. Treatment Approach

The current treatment highly depends on the gastrointestinal polyp size and number. Endoscopic polypectomy is advised for polyps that are larger than 1 cm and easily reachable by endoscopy [10,43]. Polyps that are symptomatic, enlarging or larger than 1 cm warrant a laparotomy. A “clean sweep” to remove all of the visible and reachable polyps can be attempted by the surgeon, as this can greatly reduce the need for recurrent laparotomies [44,45].

Pharmaceutical studies evaluating the use of mTOR inhibitor, rapamycin, have shown promise in polyp reduction in animal models. This suggests that rapamycin and its analogs may represent a targeted therapy for the treatment of PJS. [46] However, a Phase II study assessing the impact of everolimus on polyp and tumor growth was inconclusive, due to the small sample size and severe adverse events [47]. Other potential therapeutic agents include cyclooxygenase-2 (COX-2) inhibitors and metformin. [48,49,50] Udd et al. demonstrated a reduction in gastric polyps in a subset of PJS patients after 6 months of treatment with the Cox 2 inhibitor (celecoxib). Additionally, the activation of LKB1-AMPK pathway by metformin and its analogs was found promising in slowing the tumor onset in polyposis syndromes. Aromatase inhibitors may also play a role in the treatment of large-cell calcifying Sertoli cell tumors (LCST) in PJS patients [51]. However, a bilateral orchidectomy is usually recommended given the LCST malignant potential. Despite a few possible chemotherapeutic targets, no pharmacological prophylaxis is routinely recommended at this time.

The filtered intense pulse light (IPL) with a 590 nm filter, Q-switched ruby laser, and CO_2_-based laser have been used in the treatment of disfiguring mucocutaneous pigmentations [52,53,54,55].

## 8. Conclusions

The Peutz-Jeghers Syndrome (PJS) is an autosomal dominant neoplastic syndrome with hamartomatous polyps through the GI tract, characteristic mucocutaneous pigmentations, and an elevated lifetime cancer risk. This condition is relatively rare and requires a specialized approach to manage the elevated risks of malignancies and complications related to GI polyps. The radiologist plays a key role in the management of these patients and should have a high degree of suspicion when reviewing the surveillance studies of confirmed Peutz-Jegher syndrome patients and their relatives. Surveillance imaging protocols are not yet well-established given the prevalence of this disorder. However, some published guidelines and expert consensus should be used when managing this patient population.

## Figures and Tables

**Table 2:** NCCN adult surveillance guidelines.

Location	Lifetime Risk of Developing Malignancy, %	Surveillance Method	Interval of Surveillance	Age to Initiate Surveillance, Years
Breast	32–54	Mammogram Breast MRI Clinical breast exam	Yearly Yearly Every 6 months	30
Colon	39	Colonoscopy	Every 2–3 years	18
Stomach	29	Endoscopy	Every 2–3 years	18
Small Intestine	13	Video capsule endoscopy or CT/MRI enterography	Every 2–3 years	18
Pancreas	11–36	Endoscopic US or MRI/MRCP	Yearly	30–35
Cervix	10	Pelvic exam/ Pap smear	Yearly	18–20
Uterus	9	Pelvic exam/ Pap smear	Yearly	18–20
Ovary	18–21	Pelvic exam/ Pap smear	Yearly	18–20
Lung	7–17	NA		

**Table 3:** NCCN pediatric surveillance guidelines.

Location	Screening Targets	Surveillance Method	Interval of Surveillance	Age to Initiate Surveillance, Years
Colon Cancer Stomach	Bleeding Iron deficiency anemia	Upper endoscopy and colonoscopy	If polyps found then repeat every 2–3 years. If no polyps found, then resume at 18 y Yearly	8–10
Small Intestine	Intussusception Bleeding Iron deficiency anemia	Video capsule endoscopy or CT/MRI enterography	Every 2–3 years	8–10 Can start earlier or image more frequently if findings and symptoms warrant
Ovary	Sex cord tumor with annular tubules	Physical exam and close observation for precocious puberty	Annually	8
Testes	Sertoli cell tumors	Physical exam and close observation for feminizing changes	Annually	10
